# Redox-Regulated Mitophagy and Lysosomal Dysfunction as a Convergent Mechanism in Female Infertility: Molecular Insights and Therapeutic Perspectives

**DOI:** 10.3390/cimb48040429

**Published:** 2026-04-21

**Authors:** Charalampos Voros, Fotios Chatzinikolaou, Georgios Papadimas, Athanasios Karpouzos, Ioannis Papapanagiotou, Aristotelis-Marios Koulakmanidis, Diamantis Athanasiou, Kyriakos Bananis, Antonia Athanasiou, Aikaterini Athanasiou, Charalampos Tsimpoukelis, Maria Anastasia Daskalaki, Christina Trakateli, Nana Kojo Koranteng, Nikolaos Thomakos, Panagiotis Antsaklis, Dimitrios Loutradis, Georgios Daskalakis

**Affiliations:** 1 Department of Obstetrics and Gynecology, ‘Alexandra’ General Hospital, National and Kapodistrian University of Athens, 80 Vasilissis Sofias Avenue, 11528 Athens, Greece; thanoskarpouzosdr@hotmail.com (A.K.); aristoteliskoulak@gmail.com (A.-M.K.); diamathan16@gmail.com (D.A.); tsimpoukelischa@gmail.com (C.T.); md181341@students.euc.ac.cy (M.A.D.); thomakir@hotmail.com (N.T.); panosant@gmail.com (P.A.); gdaskalakis@yahoo.com (G.D.); 2 Laboratory of Forensic Medicine and Toxicology, School of Medicine, Aristotle University of Thessaloniki, 54124 Athens, Greece; loutradi@otenet.gr; 3 Athens Medical School, National and Kapodistrian University of Athens, 15772 Athens, Greece; dr.georgepapadimas@gmail.com (G.P.); gpapamd@hotmail.com (I.P.); kojo.k@icloud.com (N.K.K.); 4 King’s College Hospitals NHS Foundation Trust, London SE5 9RS, UK; kyriakos.bananis@nhs.net; 5 IVF Athens Reproduction Center, 15123 Maroussi, Greece; antoathan16@gmail.com (A.A.); diamathan17@gmail.com (A.A.); 6 3rd Department of Internal Medicine, Aristotle University Thessaloniki, 54124 Thessaloniki, Greece; ctrak@auth.gr; 7 Fertility Institute-Assisted Reproduction Unit, Paster 15, 11528 Athens, Greece

**Keywords:** female infertility, oxidative stress, mitochondrial dysfunction, mitophagy, lysosomal dysfunction, autophagic flux, redox signaling, organelle quality control, ovarian aging, assisted reproduction

## Abstract

Conventional hormonal and clinical models inadequately clarify the complex and diverse aspects of female infertility, resulting in poor reproductive outcomes and reduced egg viability. A growing body of research indicates that female reproductive failure is mostly due to disruptions in cellular homeostasis, especially concerning organelle quality control. Oxidative stress has emerged as a crucial mediator connecting metabolic, inflammatory, and ageing-related processes to ovarian failure, however its downstream impacts on intracellular organelle turnover remain insufficiently clarified. Our narrative review encapsulates the existing data for a unified pathogenic concept focused on the redox-regulated mitochondria–lysosome axis. We examine the interaction of oxidative stress, mitochondrial malfunction, compromised mitophagy, and lysosomal deficiency in granulosa cells and oocytes. Prolonged oxidative stress may disrupt this equilibrium, leading to defective mitochondria accumulation and impaired mitophagy. This self-perpetuating cycle may ultimately jeopardises reproductive viability and oocyte integrity. The integrated axis offers a shared molecular foundation for various infertility-related diseases, such as inadequate ovarian response, obesity-associated infertility, polycystic ovary syndrome, and ovarian ageing. Ultimately, we analyse new findings suggesting that specific antioxidant chemicals modify mitophagy and lysosomal function while also neutralising reactive oxygen species, highlighting their potential use in precision fertility treatments. Our research redefines female infertility as a condition of redox-dependent organelle quality control, thereby introducing novel avenues for identifying biomarkers, categorising patients, and targeting treatments in assisted reproduction.

## 1. Introduction

Female infertility often exhibits same clinical characteristics, but significant variations in the underlying biological pathways. A reduced ovarian reserve and hormonal dysregulation are significant diagnostic markers [1]. However, these clinical criteria insufficiently reflect the intracellular processes that ultimately dictate oocyte competency. Growing experimental and translational data indicates that modifications in subcellular quality-control systems are essential in female reproductive biology. In some instances, the disturbance of organelle homeostasis seems to manifest prior to the clinical onset of certain reproductive dysfunctions [2].

Oxidative stress is recognised as a crucial element influencing ovarian function. Reactive oxygen species (ROS) are continuously generated in ovarian tissues, principally as a result of metabolic activities, inflammatory processes, steroidogenesis in granulosa cells, and mitochondrial oxidative phosphorylation [3]. When meticulously regulated, redox signalling contributes to various critical reproductive processes, including ovulation, meiotic resumption, and follicular recruitment. However, if ROS production remains elevated or becomes excessive, it may overwhelm the body’s antioxidant defence mechanisms, potentially causing damage to lipids, proteins, and nucleic acids. Oocytes seem to be particularly susceptible to this kind of stress. Their prolonged meiotic pause and reduced transcriptional activity impede the cell’s ability to activate efficient repair mechanisms [4].

Mitochondria are the primary targets and amplifiers of oxidative stress in female reproductive cells. Oocytes contain a remarkably large number of mitochondria because they depend on oxidative metabolism for maturation, fertilisation, and the early stages of embryonic development [5]. Alterations in mitochondrial membrane potential, cristae structure, electron transport chain efficacy, and mitochondrial DNA integrity have been associated with meiotic inaccuracies, diminished developmental competence, and suboptimal oocyte quality. The defective turnover of injured organelles drives the dynamic advancement of mitochondrial dysfunction, rather than depicting it as a static aberration [6].

Mitophagy is considered a primary mechanism by which cells eliminate damaged mitochondria to maintain bioenergetic integrity. Canonical mitophagy pathways, including PINK1-Parkin-mediated ubiquitination and receptor-mediated mechanisms involving BNIP3, NIX, and FUNDC1, are linked to follicle growth and ovarian ageing [7]. In experimental settings, the genetic and pharmacological disruption of mitophagy regulators results in mitochondrial buildup, increased ROS generation, and impaired fertility. Mitophagy is not exclusively a mitochondrial process. It represents a multi-organelle mechanism that is fundamentally reliant on lysosomal functionality for its execution [8].

Lysosomes serve as hubs for metabolic signalling by integrating organelle turnover, redox equilibrium, and nutrient detection. Besides terminal degradation, they govern intracellular pH and calcium equilibrium while modulating autophagic flow via mTOR-TFEB signalling [5]. Recent results indicate that oxidative stress directly influences lysosomal biogenesis, acidity, and membrane stability. Inhibiting TFEB’s translocation to the nucleus, mTOR activation dependent on ROS diminishes the expression of lysosomal genes and impairs cellular autophagy [9]. Research indicates that the accumulation of oxidative stress products in ovarian granulosa cells impairs lysosomal activity, hence obstructing autophagic flow and diminishing the efficacy of mitophagy [10].

A common finding in female infertility is the concurrent occurrence of mitophagy start signals and enduring mitochondrial abnormalities. In some instances, mitochondria are designated for degradation but cannot be adequately eliminated, resulting in the accumulation of partially impaired organelles that exacerbate oxidative stress. The persistence of this feedback loop progressively disrupts cellular homeostasis in both granulosa cells and oocytes [11].

Oxidative stress, mitochondrial malfunction, autophagy, and mitophagy in female reproduction have been thoroughly investigated, but primarily in isolation. Limited research has examined female infertility as a result of a coordinated malfunction of mitochondria, lysosomes, and the redox axis [7]. The mechanistic understanding of common reproductive phenotypes seen in clinical disorders such as polycystic ovary syndrome, obesity-related infertility, ovarian ageing, reduced ovarian reserve, and insufficient ovarian response is limited by this fragmented viewpoint [12]. Our review analyses the current experimental and translational findings linking oxidative stress, mitochondrial dysfunction, and lysosomal impairment in female reproductive cells.

The endocrine control of follicular growth profoundly affects ovarian ageing, in conjunction with intracellular stress mechanisms. Hormonal signals, including FSH, LH, and insulin-like growth factor pathways, regulate follicle recruitment, steroidogenesis, and the metabolic functions of oocytes. These endocrine signals modify the redox environment of the follicle, influencing mitochondrial activity, autophagy, and the arrangement of the meiotic spindle. Alterations in endocrine signalling modify the metabolic and oxidative conditions of the follicular milieu with advancing age. This results in the ageing of oocytes over time, diminishing their developmental capacity. While the various components of this framework are well confirmed via experiments, their integration into a unified axis in female reproductive biology remains mostly speculative and is proposed here as a hypothesis-generating paradigm.

## 2. Materials and Methods

This narrative review is founded on a structured yet non-systematic examination of the literature concerning the molecular mechanisms underlying female infertility, oxidative stress, mitochondrial malfunction, mitophagy, and lysosomal control. A thorough search was performed in PubMed/MEDLINE, Scopus, and Web of Science to uncover pertinent experimental, translational, and clinical research published from January 2000 to January 2026. This period was chosen to include both modern developments relevant to the physiology and pathophysiology of female reproduction and foundational contributions in redox biology and organelle quality control. We used pre-defined groups of keywords and Boolean operators to search the literature. This made it possible to find relevant studies in a structured and repeatable way.

The screening procedure was conducted in stages. To exclude papers that were obviously irrelevant, we first examined the titles and abstracts. Subsequently, papers deemed potentially suitable were examined in their entirety. The focus was on their connection to redox control, mitochondrial function, mitophagy, and lysosomal pathways in female reproductive biology.

The inclusion criteria were studies that clarify the molecular, cellular, or functional dimensions of redox homeostasis, mitochondrial dynamics, autophagic flux, or lysosomal control in oocytes or granulosa cells. Both human and relevant animal or in vitro research were assessed where translational significance could be fairly inferred. The exclusion criteria included purely descriptive clinical studies lacking mechanistic interpretation, studies unrelated to female reproductive biology, and reports deficient in methodological detail to support biological conclusions.

Research offering molecular, cellular, or functional insights into the regulation of redox homeostasis, mitochondrial integrity, autophagic flux, or lysosomal competence in female reproduction was deemed appropriate for inclusion. Human research and pertinent animal or in vitro models were included, contingent upon the justifiable extrapolation of results to oocytes or ovarian somatic cells. Excluded studies included only descriptive clinical data lacking mechanistic analysis, investigations focused entirely on male infertility, and studies examining autophagy-related mechanisms irrelevant to ovarian biology. This review was narrative, hence no quantitative synthesis or formal risk-of-bias evaluation was performed.

Data were retrieved from each included paper, focusing on the experimental model, reproductive environment, molecular pathways examined, and reported effects on mitochondrial function, oxidative stress signalling, mitophagy, or lysosomal activity. Special attention was directed towards research clarifying cause-and-effect interactions, including redox-dependent modulation of autophagic and lysosomal pathways, as well as pathologically induced alterations in mitochondrial quality control. The evidence was synthesised into a narrative rather than numerical data, allowing for the amalgamation of results from many trials and clinical contexts.

As this is a narrative review, no rigorous risk-of-bias evaluation or quantitative synthesis was conducted. The emphasis was placed on biological plausibility, consistency across experimental models, and the integration of mechanistic data across different levels of reproductive physiology. This technique was used to enable the development of a unified conceptual framework rather than to provide effect-size estimates.

## 3. Oxidative Stress and Redox Homeostasis in the Female Reproductive System

Physiological reactive oxygen species alter intracellular pathways crucial for communication between follicles and oocytes, as well as for the advancement of meiosis. Hydrogen peroxide can reversibly modify cysteine residues in regulatory proteins, such as protein tyrosine phosphatases and cell cycle regulators [12,13,14,15]. This is due to the relative stability of hydrogen peroxide and its capacity for easy dispersion. The precise timing of meiotic restart and germinal vesicle dissolution in oocytes is influenced by the action of maturation-promoting hormones and the redox-dependent regulation of CDC25 phosphatases [16]. Redox-sensitive MAPK and calcium signalling pathways in cumulus cells facilitate concurrent cytoskeletal remodelling and cumulus expansion. These mechanisms are essential for oocyte competence and ovulation. At the molecular level, pathways such as NRF2-KEAP1, thioredoxin systems, and NAD^+^-dependent enzymes, including sirtuins, meticulously regulate redox signals in ovarian cells.

Maintaining redox equilibrium necessitates a robust antioxidant network comprising cytosolic, mitochondrial, and extracellular defence systems. Non-enzymatic buffers such as glutathione, thioredoxin, and NADPH-dependent reductive systems function with enzymatic antioxidants such superoxide dismutases, catalase, and glutathione peroxidases [17]. This multi-faceted defence in healthy follicles inhibits the dissemination of ROS, maintains mitochondrial membrane potential, and safeguards against oxidative damage during prolonged meiotic arrest. As transcriptional activity diminishes, the antioxidant capacity of oocytes primarily derives from the earlier phases of folliculogenesis. This illustrates their reliance on pre-existing redox resilience [18].

Chronic low-grade inflammation induces the formation of ROS by permitting the infiltration of immune cells and transmitting signals via cytokines [19]. Conversely, obesity and insulin resistance increase the availability of mitochondrial substrate and electron leakage. Environmental contaminants and endocrine disruptors exacerbate oxidative stress by impairing mitochondrial enzymes and antioxidant defences. In assisted reproductive technologies, supraphysiological gonadotropin stimulation and hyperoxic in vitro culture conditions exacerbate the redox stress on already compromised oocytes and supporting cells [19]. Clinical studies in assisted reproduction show that increased oxidative stress is associated with reduced oocyte quality and worse embryo development. Elevated oxidative stress indicators have been identified in the follicular fluid of women undergoing IVF, correlating with diminished egg quality, decreased fertilisation rates, and suboptimal embryo development.

Redox dysregulation is associated with reduced mitochondrial efficiency. With advancing age, our NAD^+^ levels decline, resulting in diminished sirtuin activity [20]. This reduces antioxidant responses and mitochondrial stress adaptation and shifts the redox balance toward a persistent pro-oxidant state [15,21].

Oxidative modifications to cardiolipin impede the formation of respiratory chain supercomplexes [21]. These alterations diminish the stability of the inner mitochondrial membrane and modify the configuration of the cristae. The loss of supercomplex integrity exacerbates mitochondrial dysfunction by diminishing the efficiency of electron transport and increasing the likelihood of reactive oxygen species leaking [22]. The gradual reduction in mitochondrial efficiency in oocytes results in diminished developmental competence [23]. Oocytes require a substantial yet finite reservoir of mitochondria to facilitate early development [23].

Oxidative stress compromises genomic stability by impacting nuclear and mitochondrial DNA in distinct manners. Damage to nuclear DNA triggers the activation of cell cycle checkpoints and repair mechanisms [24]. Mitochondrial DNA is especially susceptible because to its proximity to the electron transport chain, absence of protective histones, and limited self-repair capabilities. The accumulation of oxidative mtDNA damage exacerbates oxidative phosphorylation by altering the transcription of respiratory chain subunits and hindering mitochondrial protein synthesis. This mtDNA-related issue creates a persistent molecular signature during meiotic divisions and the initial phases of embryonic development [25].

Oxidative stress not only inflicts direct damage on molecules but also alters the intracellular signalling networks that govern metabolic adaptability and organelle quality control. Prolonged exposure to reactive oxygen species disrupts nutrient-sensing pathways, particularly those associated with mTOR signalling [26]. Oxidative stresses improperly activating mTOR inhibit TFEB’s function and impede the initiation of autophagy. They also inhibit lysosomal biogenesis. This signalling mismatch disconnects damage identification from degradative capability, complicating the removal of dysfunctional organelles and exacerbating cellular stress [27]. Redox dysregulation influences various populations of follicular cells in distinct yet interconnected manners. Granulosa cells activate adaptive antioxidant responses, including NRF2-mediated transcription of detoxifying enzymes, to maintain steroidogenesis and oocyte maintenance after initial stress exposure [28]. However, prolonged oxidative stress depletes these compensatory mechanisms, altering the secretion of paracrine substances essential for oocyte maturation, mitochondrial depolarization, and reduced activity of steroidogenic enzymes. The oocyte has limited adaptability due to its reduced transcriptional flexibility and dependence on maternal components. This results in a greater decline in its function compared to the adjacent somatic cells [14,29,30].

Despite the abundance of experimental evidence indicating that oxidative stress is associated with reproductive issues, the outcomes vary across various models. The functional significance and extent of ROS appear to be significantly influenced by the experimental system, as oxidative effects are frequently amplified in vitro conditions in contrast to the in vivo follicular environment. Furthermore, the interpretation of clinical reproductive outcomes is further complicated by the dual function of ROS as signalling molecules and agents of cellular injury.

Results from experimental models lack consistency; several studies reveal context-dependent or conflicting effects of oxidative stress, highlighting the intricacy of redox signalling in reproductive biology.

## 4. Mitochondrial Dysfunction in Oocytes and Granulosa Cells

At the ultrastructural level, the architecture of reproductive cell mitochondria is continually regulated to achieve a balance between mitigating oxidative damage and optimising energy efficiency [31,32,33]. To prevent excessive ROS production during meiotic arrest, immature oocytes typically possess mitochondria characterised by sparse and poorly developed cristae, diminished respiratory capacity, and reduced membrane potential. As follicles approach the preovulatory phase, regulated alterations in the curvature of the inner membrane and the density of the cristae enhance ATP production and respiration efficiency [34]. This remodelling requires the coordinated activity of OPA1 isoforms, cardiolipin biosynthesis, and the stability of the MICOS complex. All these elements collaborate to maintain the stability of the respiratory chain. Pathological disruption of these mechanisms, shown in metabolic diseases and ovarian ageing, results in cristae disorganisation, diminished electron transport, proton leakage, and increased vulnerability to oxidative damage [35].

Mitochondrial dynamics manage functional integrity by governing the specific cycles of fission and fusion that dictate mitochondrial distribution, content mixing, and stress segregation. By enabling the equilibration of mitochondrial proteins, metabolites, and mtDNA nucleoids, fusion processes mediated by MFN1, MFN2, and OPA1 alleviate localised abnormalities [36]. DRP1-dependent fission facilitates the segregation of damaged mitochondrial components for quality control purposes. When these processes malfunction, whether due to excessive fragmentation or insufficient fission, the stability of the mitochondrial network in reproductive cells is jeopardised [37]. Phosphorylation, SUMOylation, and redox-dependent nitrosylation directly influence DRP1 activity. This indicates a clear correlation between mitochondrial dynamics and oxidative as well as metabolic states in cells. An imbalance in these dynamics results in an irreversible buildup of defective organelles in oocytes, where mitochondrial biogenesis is restricted after growth completion [37].

Severe bioenergetic insufficiency indicates mitochondrial malfunction that transcends structural alterations and directly impacts reproductive capability. A combination of diminished oxidative phosphorylation complex activity, inadequate supercomplex formation, and suboptimal preservation of the electrochemical proton gradient impedes ATP synthesis in cells [38]. Oocytes exhibit heightened sensitivity to fluctuations in ATP levels, as spindle construction, kinetochore attachment, chromosomal congression, and cytoplasmic maturation all require a meticulously regulated energy supply. Even minimal ATP depletion can destabilise microtubule dynamics and increase the likelihood of meiotic nondisjunction [39]. The inefficiency of mitochondria in granulosa cells impedes steroidogenesis by restricting NADPH availability, blocking cholesterol transport through the StAR route, and diminishing the activity of mitochondrial P450 enzymes, which indirectly undermines oocyte maturation.

A frequently overlooked aspect of susceptibility in female reproductive cells is the integrity of mitochondrial DNA [40]. Mitochondrial DNA is especially vulnerable to oxidative damage owing to its closeness to the electron transport chain and its deficiency of effective repair mechanisms and histone protection. Point mutations, deletions, and alterations in copy number can accumulate, complicating the transcription and translation of the respiratory chain subunits within the mitochondrial genome [41]. These deficiencies increase reactive oxygen species, alter the stoichiometry of electron transport, and facilitate electron leakage. Due to the infrequent turnover of oocytes, faulty mtDNA persists within them and is transmitted to the embryo. This influences the quality of the blastocyst, the dynamics of early cleavage, and potentially the long-term health of the progeny [4].

Mitochondrial failure induces several adaptive and unfavourable stress responses, altering the destiny decisions of follicular cells. When AMPK is activated due to insufficient energy, metabolism shifts towards catabolic pathways and ceases anabolic processes such as protein and fat synthesis [42]. Prolonged activation of AMPK signalling inhibits follicular growth and disrupts differentiation processes. Conversely, short-term stimulation may sustain cellular viability. Mitochondrial stress simultaneously initiates the unfolded protein response, altering nuclear transcription to enhance proteostasis and chaperone expression [26]. Prolonged stimulation of these pathways leads to maladaptive outcomes, such as functional decline and symptoms characteristic of cellular senescence. Mitochondria serve as crucial signalling centres that connect metabolic conditions with routes for survival and apoptosis [43]. When impaired mitochondria emit reactive oxygen species, cytochrome c, and other pro-apoptotic signals, the threshold for both apoptotic and non-apoptotic cell death diminishes. The depolarisation of granulosa cell mitochondria increases the likelihood of follicular atresia by initiating intrinsic apoptotic pathways. Conversely, oocytes frequently avoid apoptosis despite mitochondrial malfunction, leading to sublethal damage marked by a lack of developmental competence. This distinction facilitates the survival of oocytes that possess a normal morphology yet have suboptimal functionality [44]. Studies on human IVF indicate that mitochondrial integrity is crucial for oocyte competency [39]. Oocytes exhibiting altered mitochondrial membrane potential and aberrant mitochondrial distribution are associated with diminished fertilisation potential and suboptimal embryo development.

Membranes associated with mitochondria facilitate calcium signalling, lipid transfer, and coordination between the endoplasmic reticulum and mitochondria [45]. Alterations in these contact points disrupt bioenergetic control, exacerbate oxidative stress, and diminish calcium buffering. Dysregulated calcium flux in reproductive cells influences signal transduction pathways critical for follicular growth, steroidogenesis, and meiosis progression. The metabolic and redox imbalance is exacerbated by analogous crosstalk issues involving lysosomes and peroxisomes [46,47]. Granulosa cells initially exhibit greater resistance to mitochondrial stress due to their ability to proliferate and adapt their metabolic processes. However, prolonged mitochondrial dysfunction depletes these adaptive mechanisms, resulting in diminished steroidogenesis, alterations in growth factor production, impaired gap junction communication, and a reduction in metabolic support for oocytes [6].

The bioenergetic capacity of mitochondria is essential during meiotic maturation, since spindle formation, chromosomal alignment, and cytoplasmic maturation need significant ATP for optimal operation.

## 5. Mitophagy as a Quality Control Mechanism in Female Reproduction

The PINK1-Parkin signalling axis functions as a molecular switch responsive to alterations in membrane potential. Identifying damage to mitochondria is of paramount importance [48,49,50]. The TOM/TIM complexes typically transport PINK1 into polarised mitochondria, where PARL subsequently cleaves it into smaller fragments, ultimately resulting in its degradation in the cytosol [51]. When the loss of mitochondrial membrane potential halts this import process, full-length PINK1 can accumulate on the outer mitochondrial membrane. PINK1 phosphorylates ubiquitin at Ser65 to bind and activate cytosolic Parkin, initiating a feed-forward amplification loop [52]. Activated Parkin facilitates the polyubiquitination of many outer mitochondrial membrane proteins, such as mitofusins, Miro, and VDAC, to immobilise injured mitochondria and inhibit their reintegration into the healthy mitochondrial network [53].

Parkin-mediated mitophagy is intricately associated with cytoskeletal and trafficking mechanisms, as well as ubiquitination, to ensure the spatial confinement of injured mitochondria [54]. The deceleration of Parkin-dependent degradation of Miro proteins impedes mitochondrial motility along microtubules, so effectively sequestering damaged organelles and facilitating their encapsulation by autophagosomes. The spatial regulation of mitochondrial location is crucial in oocytes, since mitochondria congregate around spindle and cortical regions [55].

Receptor-mediated mitophagy pathways offer a supplementary and frequently primary mechanism for mitochondrial turnover in reproductive organs, operating in conjunction with the PINK1-Parkin axis. BNIP3, NIX, and FUNDC1 are located in the outer mitochondrial membrane [7]. They engage directly with LC3 via LC3-interacting areas without undergoing ubiquitination. Due to the constant fluctuations in their phosphorylation status, food availability, and hypoxia-inducible factors, these receptors can rapidly adjust to alterations in the follicular environment. Receptor-mediated mitophagy facilitates metabolic reprogramming during follicular maturation and luteal differentiation by eliminating mitochondria optimised for oxidative metabolism and substituting them with those more suited for steroidogenesis or glycolytic coupling. When these receptors malfunction, granulosa cell differentiation is impaired, and mitochondrial remodelling is decelerated [56].

The onset of mitophagy and mitochondrial dynamics are intricately linked, as damaged mitochondria must be segregated from the functional network for selective elimination. Mitochondrial fragments produced by DRP1-mediated fission can be evaluated separately for membrane potential, ROS generation, and protein integrity [57]. Impaired fission complicates the segregation of damaged components and results in the accumulation of various mitochondrial types [58]. Conversely, hyperactivation of fission without compensating mitophagy results in excessive mitochondrial fragmentation and energy depletion. Post-translational changes in DRP1, such as phosphorylation at Ser616 or Ser637 and redox-dependent S-nitrosylation, link oxidative stress and mitophagy efficacy at a molecular level [59,60,61].

Functional lysosomes capable of acidification, hydrolysis, and membrane fusion are essential for the removal of damaged mitochondria, even when they are accurately identified and isolated. Oxidative stress-induced activation of mTOR signalling in ovarian cells suppresses the transcription of lysosomal enzymes, membrane proteins, and v-ATPase subunits by hindering autophagy start and impeding the nuclear translocation of TFEB. Impaired lysosomal acidification may result in the buildup of autophagolysosomes with incompletely digested mitochondrial debris, thereby obstructing mitochondrial breakdown [62,63]. Cell-type-specific limitations additionally influence the outcomes of mitophagy in the ovarian follicle. Granulosa cells can temporarily compensate for mitochondrial injury by enhancing their mitophagic flux and possessing a greater lysosomal reserve [64]. Oocytes exhibit limited capacity for upregulation and depend on a pre-existing pool of lysosomes and autophagic components.

When mitochondrial damage surpasses the capacity of mitophagy to eliminate it, granulosa cells mostly engage intrinsic apoptotic pathways [56]. This results in follicular atresia and a reduction in the follicle reserve. In oocytes, apoptosis is meticulously managed; in contrast, mitochondrial dysfunction results in functional inadequacy, evidenced by early embryonic arrest, diminished fertilisation rates, and altered calcium oscillations. The accumulation of dysfunctional mitochondria and the progressive decline in reproductive capacity are both attributable to a disruption at any stage in this sequence, particularly at the level of lysosomal functionality [65]. Notably, analogous pathways have been identified in human granulosa cells acquired during IVF cycles, whereby compromised mitophagy and mitochondrial dysfunction correlate with diminished oocyte competence and subpar reproductive outcomes [65].

It is crucial to acknowledge that a substantial portion of the current body of knowledge on mitophagy and lysosomal regulation is derived from experimental frameworks that are either non-reproductive or scrupulously regulated. The extent to which these mechanisms operate in human oocytes, which exhibit unique metabolic and transcriptional constraints, remains to be thoroughly understood. This limits the direct conversion of mechanistic discoveries into clinically significant interpretations. Moreover, a significant portion of the existing research is derived from in vitro or animal models, which may inadequately reflect the distinct metabolic and developmental limitations of human oocytes.

## 6. Lysosomal Function and Autophagic Flux

The integrity of lysosomes in female reproductive cells is critically important, as oocytes must maintain cytoplasmic competence for decades without cell division or significant organelle renewal [66]. Oocytes are particularly vulnerable to cumulative lysosomal dysfunction, as their lysosomal capacity is primarily developed during oogenesis and thereafter sustained with limited replenishment. Granulosa cells exhibit mitotic activity. They experience various metabolic alterations throughout follicular development, ovulation, and luteinization. Thus, lysosomal–autophagic systems are frequently employed to enhance lipid metabolism, organelle turnover, and steroidogenic remodelling [67].

The CLEAR (Coordinated Lysosomal Expression and Regulation) gene network, primarily regulated by TFEB, orchestrates lysosomal development and cellular autophagy at the transcriptional level [68]. TFEB dynamically modifies lysosomal capacity by incorporating cellular stress signals, energy state, and nutrition supply. In nutrient-abundant situations, lysosome-associated mTORC1 phosphorylates TFEB, thereby retaining it in the cytoplasm. TFEB dephosphorylates and translocates to the nucleus in response to oxidative stress, hunger, or increased autophagy requirements [69]. In that area, it induces the expression of genes associated with autophagy, membrane proteins, trafficking regulators, and lysosomal hydrolases. Ovarian cells must meticulously regulate TFEB activity over time to align lysosomal output with the evolving requirements of cytoplasmic remodelling, steroidogenesis, and mitochondrial turnover throughout follicular development.

Chronic oxidative stress significantly impairs this regulatory axis by aberrantly increasing mTORC1 signalling. Prolonged exposure to ROS in the ovarian microenvironment, resulting from metabolic overload, mitochondrial dysfunction, environmental pollutants, or inflammatory signalling, activates redox-sensitive kinases upstream of mTORC1, causing persistent phosphorylation of TFEB despite cellular stress [70]. The erroneous mTORC1 activity, which dissociates lysosomal biogenesis from autophagic requirements, gradually depletes the lysosomal reserve. Consequently, the degradative capacity of oocytes progressively declines. Granulosa cells initially augment autophagic flow, but prolonged stress exhausts this adaptive capacity, leading to metabolic instability and impaired organelle clearance [71].

The vacuolar H^+^-ATPase (v-ATPase) complex performs functions beyond mere transcription regulation. It also facilitates the acidification of the lysosomal lumen, which is essential for lysosomal functionality. Acidification stimulates cathepsins and other hydrolases essential for the degradation of autophagic substrates such as mitochondria, protein aggregates, and lipid droplets [72]. Oxidative stress impairs v-ATPase construction and function in ovarian cells through two mechanisms: inducing lipid peroxidation of lysosomal membranes and causing oxidative alteration of proton pump subunits. Inadequate acidification results in the accumulation of partially digested organelles, hence impeding the rate of proteolytic processes and diminishing the efficacy of degradation [73]. Impaired lysosomal acidification during mitophagy hinders mitochondrial breakdown at an advanced stage, allowing defective mitochondrial pieces to remain and persist in generating ROS, thus increasing cellular stress.

The integrity of the lysosomal membrane is a significant aspect that influences autophagic flow. Lysosomal membranes are particularly susceptible to oxidative damage due to their lipid composition and exposure to redox-active iron [74]. Lysosomal membrane permeabilization (LMP), induced by oxidative stress, results in the leakage of cathepsins, calcium, and other luminal constituents into the cytosol. Prolonged membrane destabilisation irreversibly impairs lysosomal function and enhances apoptotic signalling, while transitory and localised lysosomal membrane permeabilization may trigger adaptive stress responses [75]. LMP promotes follicular atresia and reduces the apoptotic threshold in granulosa cells. Lysosomal leakage causes prolonged cytoplasmic stress and functional damage in oocytes, rather than instantaneous cell death, where apoptotic processes are meticulously regulated [44].

The effective operation of autophagic flux necessitates meticulous coordination between the fusion of autophagosomes and lysosomes. This process is facilitated by SNARE proteins, Rab GTPases, tethering complexes, and alterations in the cytoskeleton [76]. Oxidative stress modifies the formation of the SNARE complex and the stability of microtubules in ovarian cells, hence influencing the rate of fusion. Impaired fusion results in the accumulation of autophagosomes containing undigested cargo. This is a prevalent indicator of infertility. The accumulation of these stalled intermediates further impedes the degradation processes and hinders the functionality of the autophagy apparatus, exacerbating cellular stress rather than alleviating it [77].

Lysosomes serve as crucial intracellular signalling centres that integrate metabolic and stress signals to orchestrate anabolic and catabolic responses. Cells regulate protein synthesis, lipid metabolism, and autophagy via lysosome-associated mTORC1 signalling in accordance with dietary availability [78]. This signalling axis must be meticulously regulated to sustain equilibrium among proliferation, differentiation, and quality control in female reproductive cells. Oxidative stress alters lysosomal signalling and induces aberrant mTORC1 activation by modifying lipid composition, calcium flow, and amino acid sensing. When this maladaptive signalling inhibits the initiation of autophagy and exacerbates lysosomal insufficiency, it triggers a continuous loop of diminished organelle turnover [79].

Lysosomal calcium signalling is a crucial aspect of lysosomal regulation that is sometimes overlooked yet directly influences women’s fertility. Lysosomes function as intracellular Ca^2+^ reservoirs, releasing calcium via channels such as TRPML1 and TPCs in response to metabolic cues [80]. The release of lysosomal Ca^2+^ is essential for the fusion of autophagosomes and lysosomes, the localisation of lysosomes, and the activation of subsequent signalling cascades. Oxidative stress impairs lysosomal calcium homeostasis by modifying channel functionality and luminal ion equilibrium, hence obstructing fusion events and inhibiting autophagic flux. Impaired lysosomal Ca^2+^ signalling in oocytes is directly linked to diminished developmental potential, as it disrupts calcium-dependent processes essential for meiotic progression and fertilisation competence [46].

Lysosomes translocate between the perinuclear and peripheral areas in response to nutritional and energy inputs to optimise degradation and signalling processes [81]. The placement is regulated by microtubule-mediated transport and small GTPases that react to AMPK and mTOR signals. Chronic oxidative stress impedes lysosomal mobility in ovarian cells, leading to aberrant clustering and diminished accessibility to autophagic cargo. Mislocalization of lysosomes impedes effective organelle clearance and contact with damaged mitochondria in oocytes, which are marked by a highly polarised cytoplasmic structure [82,83].

Inter-organelle communication exacerbates the issues arising from lysosomal dysfunction. Lysosomes engage in bidirectional communication with mitochondria, the endoplasmic reticulum, and peroxisomes via specialised contact sites that regulate calcium signalling, lipid exchange, and redox equilibrium [84]. Disruption of lysosome–mitochondria interaction sites exacerbates oxidative stress and impairs calcium-dependent regulation of mitochondrial metabolism. Peroxisomal interactions regulate redox homeostasis, while interactions with the endoplasmic reticulum alter cellular stress responses and protein folding mechanisms. When these communication networks are disrupted, the capacity of oocytes to undergo meiosis and initiate early embryonic development is compromised [85]. When lysosomes malfunction, mitophagy transitions from a protective quality control process to a pathogenic catalyst of cellular failure. Despite the accurate tagging and sequestration of mitochondria by autophagosomes, incomplete lysosomal breakdown results in the buildup of mitochondrial remains. These remnants exacerbate redox imbalance and energy deficiency by increasing reactive oxygen species and metabolic stress. Mitochondrial dysfunction is exacerbated by defective mitophagy, accelerating the decline of reproductive ability [86].

Cell-type-specific constraints significantly influence the impact of lysosomal dysfunction on the ovarian follicle. Granulosa cells can temporarily enhance their lysosomal capacity under stress due to increased lysosomal plasticity [87]. This reserve is burdened by prolonged oxidative and metabolic stress, resulting in lysosomal depletion, compromised steroidogenesis, and disruption of paracrine signalling to the oocyte. Conversely, oocytes can only enlarge their lysosomes to a limited extent under stress and depend on a restricted reservoir of lysosomes. Lysosomal failure consequently adversely impacts oocyte mitochondrial quality, cytoplasmic maturation, and developmental competence. Lysosomal malfunction represents a significant pathogenic connection between female infertility, oxidative stress, and compromised mitophagy. Lysosomal dysfunction impairs cellular homeostasis by limiting autophagic flux, destabilising intracellular signalling, and hindering inter-organelle communication. The integrity of lysosomes is a vital factor influencing the longevity and reproductive success of female gametes, requiring ongoing organelle quality monitoring for continued operation [88].

## 7. Integrated Mitochondria–Lysosome-Redox Axis: Molecular Architecture, Signalling Microdomains, and Failure Thresholds

At the molecular level, the proposed mitochondria–lysosome-redox axis may be conceptualised as a decentralised control system rather than a linear stress-response mechanism [89,90,91]. To facilitate comprehension, the components of this axis can be organised into three interconnected levels: (i) redox signalling and metabolic input, (ii) mitochondrial quality control and the initiation of mitophagy, and (iii) lysosomal processing and the conclusion of autophagy. The proposed axis can be divided into three levels of hierarchy to facilitate the understanding of the concept: (i) upstream drivers, which are primarily redox imbalance and metabolic stress; (ii) intermediate regulatory processes, which encompass mitochondrial dynamics and the initiation of mitophagy; and (iii) downstream execution mechanisms, which are primarily lysosomal function and autophagic flux.

The energy-redox coupling inside mitochondrial microdomains serves as a fundamental organising principle of the axis. Mitochondria are not homogeneous metabolic entities; rather, specific mitochondrial subpopulations within the oocyte or granulosa cell exhibit distinctive membrane potentials, substrate affinities, and redox outputs [92]. These microdomains generate localised redox signals that modify adjacent proteins in a manner reversible by oxidation, nitrosylation, or glutathionylation. Such alterations modify enzyme functionality and protein interactions without inducing widespread oxidative damage. For this messaging to be precise, it must be spatially proximate. When the integrity of mitochondrial microdomains is compromised, redox signals disseminate beyond their designated targets, transforming localised regulatory signals into widespread cellular stress [93,94,95].

Lysosomal signalling integration primarily occurs at the lysosomal surface, serving as a molecular decision-making platform. The Ragulator-Rag GTPase complex, v-ATPase, and additional scaffolding proteins form a coincidence-detection system that integrates the availability of amino acids, the ATP to AMP ratio in the cytosol, and the redox state [96]. Mitochondrial failure significantly alters the local concentrations of metabolites, particularly acetyl-CoA, NAD^+^, and amino acids derived from glutamine. These alterations directly influence the nucleotide loading of Rag GTPase [97]. This allows lysosomes to assess mitochondrial metabolic capability regardless of external food availability, a vital characteristic in the follicular milieu where systemic and local nutrition signals often differ.

The redox control of lysosomal signalling transpires through the selective oxidation of regulatory cysteines in regulator components and mTORC1-associated phosphatases. These alterations do not cease signalling [98]. They modify its hysteresis, hence affecting the threshold at which mTORC1 ceases interaction with the lysosomal membrane. In adaptive stages, this barrier remains low, facilitating the body’s rapid transition between anabolic and catabolic modes. Prolonged oxidative circumstances elevate the threshold, maintaining constant mTORC1 connection and inhibiting autophagic initiation despite the presence of organelle damage. This represents a molecular point of irrevocability on the axis [99,100,101].

Mitochondria and lysosomes interact at specific sites that function as high-fidelity signalling routes independent of cytosolic diffusion. These contact locations facilitate bidirectional calcium flow, lipid mobility, and localised kinase activation [89]. The release of calcium from lysosomes via TRPML1 channels directly influences mitochondrial dehydrogenase activity, while mitochondrial calcium intake impacts the positioning and motility of lysosomes. Redox imbalance disrupts the chemical frameworks that maintain these contact sites, resulting in diminished coherence of the signals rather than their entire cessation. This results in asynchronous organelle activity, wherein mitochondria and lysosomes respond to stress at disparate intervals, hence diminishing the efficacy of quality control [102,103]. Each instance of malfunction results in molecular damage, such as oxidised lipids, altered proteins, and misfolded hydrolases, which subtly diminish the efficiency of subsequent processing. Gradually, these minor losses diminish the system’s stability until it attains a critical threshold. Subsequent damage leads to a rapid decline in autophagic flux. This temporal integration clarifies the phenomenon of sudden acceleration in reproductive decline, rather than a progressive trend [104].

Oocyte-specific constraints further delineate these levels. Oocytes, in contrast to somatic cells, are unable to dilute damaged components during division and can only synthesise new lysosomes to a limited extent [105]. Consequently, the axis consistently operates near its threshold. Subtle abnormalities that are mitigated in granulosa cells accumulate imperceptibly in oocytes, reducing adaptive capacity long before clinical dysfunction becomes apparent. The asymmetry accounts for the appearance of good follicular growth, but the oocyte’s functional capacity may be deficient. A significant molecular attribute of the integrated axis is signal memory, which is preserved by post-translational changes. Oxidative and metabolic damage inflicts enduring alterations on kinases, phosphatases, lipid-modifying enzymes, and scaffolding proteins. These modifications alter the fundamental signalling states and the speed at which the body reacts to novel stimuli. In reproductive cells, this memory accumulates throughout the reproductive lifespan, hence diminishing the adaptation window of the axis. Thus, insults that are tolerated during early reproductive years result in swift failure in later life [106].

The failure of the integrated axis does not provide homogeneous cellular effects. The impairment of lysosomal processing in granulosa cells diminishes apoptotic thresholds, hence promoting follicular atresia. In oocytes, apoptosis is actively suppressed, resulting in a failure to achieve functional uncoupling [44]. This disorder is characterised by desynchronised ATP availability, aberrant calcium signalling, altered redox gradients, and compromised organelle inheritance during early development. These flaws are inconspicuous and sometimes overlooked during morphological evaluations, however they significantly impact an individual’s developmental potential. The mitochondria–lysosome-redox axis operates as a multistable network defined by specific adaptive and maladaptive states at the systemic level. Threshold-dependent switches, rather than steady parameter drift, govern the transition between states. This architecture clarifies individual differences in reproductive ageing and infertility risk, as slight variations in baseline organelle quality or stress exposure determine whether cells remain within an adaptive basin or experience irreversible failure [107].

This approach effectively reconciles the mechanical convergence of diverse infertility-related diseases. Metabolic diseases, endocrine disruptors, environmental toxins, and chronological ageing present unique proximal insults but converge at common molecular bottlenecks within the axis of lysosomal processing capacity, redox-sensitive signalling thresholds, and organelle interface integrity [108]. These choke points are not fixed deficiencies; they are dynamic vulnerabilities that emphasise time and cumulative exposure rather than isolated incidents. The integrated axis model indicates that therapies targeting isolated nodes are unlikely to restore system-level stability from a therapeutic standpoint. Antioxidants may transiently diminish redox noise. They cannot reinstate signalling thresholds if lysosomal processing capability remains compromised. Conversely, promoting mitochondrial activity without enhancing degradative processes may accelerate the accumulation of damage. For efficacy, the intervention must re-synchronise the components of the axis to ensure that mitochondrial output, redox signalling, and lysosomal clearance are harmonised [109].

In assisted reproduction, this axis undergoes considerable disruption because to supraphysiological stimulation, altered metabolic settings, and in vitro culture conditions. These interventions impose significant stress on a system that is already near its threshold, increasing the likelihood of irreversible state changes. Understanding the interplay between ART procedures and axis dynamics establishes a mechanistic basis for improving stimulation tactics and culture conditions focused on sustaining organelle coordination rather than emphasising short-term output maximisation. To facilitate the conceptual integration of the aforementioned molecular architecture, Table 1 delineates the primary components of the axis and their functional roles in reproductive cells.

**Figure 1:**
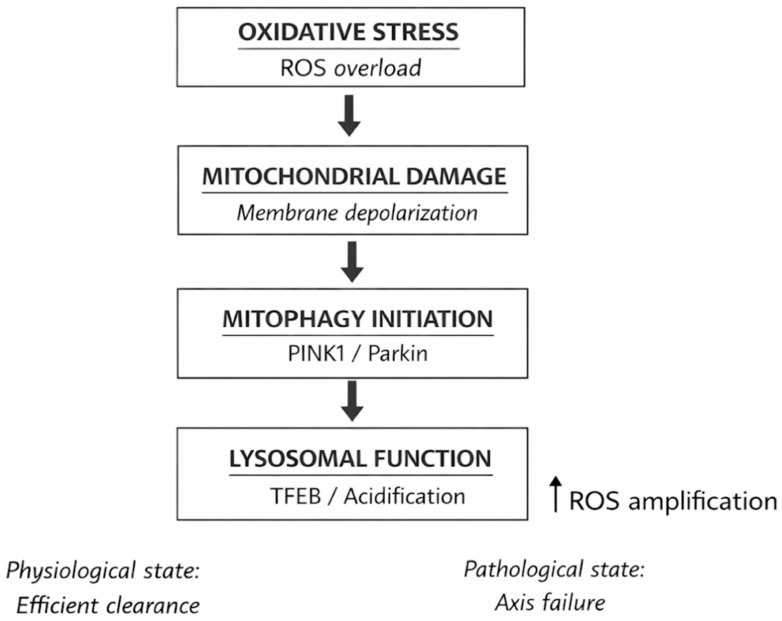
The redox-mitochondria–lysosome axis in female reproductive cells. Oxidative stress makes mitochondria stop working right. Damaged mitochondria activate mitophagy pathways through PINK1-Parkin signalling and receptor-mediated mechanisms. For effective mitochondrial clearance, lysosomal degradation must be regulated by TFEB-mTOR signalling and lysosomal acidification. If this quality-control system does not work, mitochondria that do not work properly buildup, oxidative stress rises, and the ability of oocytes to reproduce and their potential to do so slowly goes down.

The main molecular subsystems that make up the redox-mitochondria–lysosome axis in female reproductive cells are summarised in this table. Redox homeostasis, autophagic flux, mitochondrial quality control, and lysosomal degradation are all connected by the axis, which functions as an integrated control network. Oocyte competence and follicular function gradually deteriorate when any node is disrupted. Figure 1 summarises the axis in female reproduction. This integrated paradigm mostly relies on the convergence of mechanistic discoveries from many experimental contexts, with direct confirmation in human reproductive cells being restricted.

This framework has the potential to clarify the persistent discrepancies observed between traditional morphological evaluation and actual reproductive outcomes from a clinical perspective. Subclinical mitochondrial and lysosomal dysfunction may be present in oocytes and embryos that exhibit normal morphological characteristics, but this dysfunction is not detectable by conventional evaluation criteria. This may partially account for the variations in the efficacy of implantation, embryo development, and fertilisation rates in assisted reproductive technologies.

Despite robust mechanistic data, the direct application of these pathways to clinical infertility remains limited, with just a few of studies demonstrating consistent associations between these pathways and reproductive outcomes in people. To bridge this gap, it is essential to conduct meticulously designed human studies that link molecular discoveries to clinically significant outcomes such as embryo growth, oocyte quality, and pregnancy results.

## 8. Disease-Specific Molecular Disruption of the Mitochondria-Lysosome-Redox Axis

The destabilisation of the mitochondria–lysosome-redox axis adheres to disease-specific molecular pathways that signify distinct initiating insults, signalling biases, and temporal dynamics, even though it embodies a conserved regulatory framework. Infertility-related disorders differ significantly in both the extent of axis disruption and the molecular node at which coordination first fails. This distinction has a big effect on oocyte competence, the ability to reverse dysfunction, and how well a treatment works.

### 8.1. Polycystic Ovary Syndrome: Androgen-Driven Rewiring of Mitochondrial-Lysosomal Signalling

The primary destabilisation of the mitochondria–lysosome-redox axis in polycystic ovarian syndrome appears to arise, at least in part, from hormone-induced reconfiguration of intracellular signalling hierarchy rather than inherent mitochondrial susceptibility [110]. Chronic androgen excess does not directly result in organelle failure; instead, it creates a sustained distortion in granulosa cell signalling networks, modifying the interpretation of metabolic and quality-control signals. Understanding why PCOS may be a potentially reversible kind of axis disruption necessitates grasping this distinction [111]. Activation of the androgen receptor modifies the transcriptional and post-translational regulation of enzymes that govern substrate entrance into the tricarboxylic acid cycle at the mitochondrial level. A shift in the redox equilibrium of the electron transport chain occurs when fatty acid oxidation becomes less adaptable and glycolytic intermediates gain prominence [112]. This metabolic alteration is significant, as it facilitates the dissemination of ROS signals by gradually augmenting electron leakage at certain respiratory complexes, without immediately halting ATP generation. Due to their lack of a precise location, these signals contribute to low-intensity redox noise rather than successfully initiating mitophagy [113]. Androgens influence mitochondrial dynamics by altering the fission-fusion machinery via kinase, alongside their impact on metabolism. Continuous stimulation of AKT- and ERK-dependent pathways modifies the phosphorylation status of mitochondrial shape proteins, leading to fragmentation without initiating compensatory fusion. The modified metabolite channelling and diminished cristae architecture of this fragmented mitochondrial network further diminish signal specificity. Mitochondrial variability rises in this situation; nevertheless, quality control mechanisms inadequately distinguish between functional and non-functional organelles [114].

Lysosomal dysfunction in PCOS mostly results from signalling rather than structural causes. Androgen-augmented PI3K-AKT signalling maintains mTORC1’s association with the lysosomal membrane, irrespective of cellular energy levels [115]. This erroneous prolongation of anabolic signalling halts lysosomal adaptation, despite the presence of stress signals in the mitochondria. The lysosomal compartment is significant as it maintains its structure, however it cannot alter its efficacy in degradation when necessary. Mitophagy initiation signals are generated, although their execution remains incomplete. The impact of androgens on lysosomal localisation and mobility is a largely unexamined aspect of the dysregulation associated with PCOS. Cytoskeletal remodelling, occurring subsequent to androgen receptor activation, alters microtubule dynamics, impeding lysosomal transport towards regions abundant in mitochondria. Despite the presence of the autophagic machinery, this spatial decoupling complicates the clearance of mitochondria by diminishing the likelihood of effective autophagosome–lysosome interactions [116].

In PCOS, redox signalling displays a unique qualitative signature. Cells undergo a continuous, low-grade redox imbalance that gradually modifies signalling thresholds, compensating for high-amplitude oxidative stress. Redox-sensitive phosphatases extend kinase signalling downstream of growth factor and androgen receptors by progressively transitioning to oxidised, inactive forms [117]. The axis exhibits diminished sensitivity to mitochondrial distress signals with time due to the self-reinforcing nature of the signalling bias in a feed-forward loop.

Excess androgen reinforces this maladaptive condition at the chromatin level through metabolic-epigenetic coupling. Modified mitochondrial metabolism affects the levels of acetyl-CoA and NAD^+^ within cells, hence influencing sirtuin activity and histone acetylation [118]. These epigenetic modifications effectively inscribe signalling bias into the chromatin architecture by promoting transcriptional programmes that enhance glycolytic metabolism and suppress stress-adaptive responses. This epigenetic reinforcement elevates the standard for restoring normal axis dynamics [119].

This rewiring exerts a significant albeit indirect influence on the oocyte. Granulosa cells in PCOS obscure the variation in organelles by retaining their capacity for growth and steroidogenesis. The oocyte experiences fluctuating redox conditions and inadequate metabolic support, hindering mitochondrial maturation without inflicting significant damage [120]. As a result, oocytes with normal appearance but diminished developmental capacity are produced. The PCOS phenotype does not exhibit axis collapse owing to injury; rather, it demonstrates an issue with signal interpretation. Lysosomes retain their capacity for degradation, mitochondria continue to emit distress signals, and redox signalling remains within permissible thresholds [121]. Androgen-induced hierarchy inversion disrupts the coordination of these signals. The dominance of nutrition and growth signals over quality-control indicators hinders efficient coordination. This mechanistic profile clarifies several clinical observations [122]. Inferior egg quality in PCOS indicates poor cellular cooperation, although an elevated follicle count suggests ongoing signalling for growth among the cells. Rebalancing signalling priorities allows the axis to re-enter an adapted state without requiring structural organelle regeneration, clarifying why metabolic or hormonal therapies may help certain patients restore fertility.

### 8.2. Obesity-Associated Infertility: Molecular Saturation of Mitochondrial and Lysosomal Quality-Control Circuits

The mitochondria–lysosome-redox axis is impaired in obesity-related infertility due to the molecular saturation of quality-control mechanisms, rather than signalling bias or fundamental organelle damage. Chronic nutritional excess forces continuous changes in metabolic and degradative pathways, gradually undermining regulatory mechanisms that typically distinguish between pathogenic stress and adaptive remodelling [123]. Thus, the condition is defined by a reduction in decision fidelity at several molecular nodes rather than by inherent damage. Prolonged exposure to high levels of lipid-derived substrates modifies the kinetics of β-oxidation and the relationship between electron transport and substrate oxidation in the mitochondria. The accumulation of surplus acyl-CoA species in the inner membrane and mitochondrial matrix interferes with critical dehydrogenases, altering the ratios of NADH to FADH_2_ production [124]. This renders electron transfer pathways that are less effective at proton pumping more significant by directing electron input into the respiratory chain towards pathways associated with complex II and ETF. The result is a persistent change in redox pressure at particular respiratory complexes, especially in regions susceptible to electron backflow, rather than an acute ATP deficiency [121].

These alterations disrupt the assembly kinetics of respiratory supercomplexes. For supercomplex stability, it is essential to maintain the appropriate quantities of each complex and lipid cofactors, including cardiolipin. Excessive lipids alters the composition of acyl chains and the enzymes involved in cardiolipin remodelling [125]. This results in supercomplexes remaining stationary for a shorter duration and undergoing more rapid turnover. This destabilisation enhances random electron leakage without sufficiently depolarising the membrane to activate conventional mitophagy sensors. Mitochondria evade elimination by quality control by remaining in a biochemically inefficient yet bioenergetically viable condition.

The saturation of protein import and folding mechanisms worsens mitochondrial proteostasis [126]. Nutrient-responsive transcriptional programmes produce excessive nuclear-encoded mitochondrial proteins, overwhelming mitochondrial chaperones and TOM/TIM translocase systems. The accumulation of proteins in partly folded states exerts increased stress on mitochondrial proteases such as LONP1 and CLPP. As these proteases approach their peak activity, their capacity to degrade damaged proteins in a targeted manner diminishes. This allows misfolded components to accumulate and obstruct enzymatic complexes. This exemplifies a proteostatic bottleneck that diminishes the accuracy of mitochondrial signalling without compromising the integrity of the organelle [127].

For mitophagy to initiate, signs of molecular damage must persist for an extended duration. Mitochondrial dysfunction in obesity is intermittent. Mitochondria oscillate between stressed and almost normal states due to variations in substrate availability and hormonal conditions, which impede the establishment of persistent markers like ubiquitin chains or receptor engagement necessary for the initiation of destruction [128]. Owing to this temporal instability, malfunctioning mitochondria can reintegrate into the active network by interfering with mitophagic decision-making. In contrast to structural damage, lysosomal systems undergo a simultaneous saturation due to an incessant requirement for degradation [86]. Excessive prolonged consumption elevates the baseline autophagic flux, necessitating lysosomes to operate at their maximum capacity for an extended duration. Lysosomal acidification emerges as the constraining force in these scenarios. The v-ATPase regulates a stable pH in the luminal environment by utilising ATP to transport protons. However, prolonged operation results in partial uncoupling, diminishing its efficacy in acidifying the environment [129]. Minor alterations in luminal pH can significantly diminish the efficacy of acid hydrolases in degrading proteins and organelles. In these scenarios, hydrolase kinetics transition from substrate-limited to enzyme-limited circumstances. Enzymes associated with lipid hydrolysis are more prone to stabilisation due to the constant availability of substrates. Nucleases and proteases have a reduced turnover and are more susceptible to alterations induced by oxidative stress. This selective stabilisation alters the lysosome’s capacity for degradation, rendering lipid processing more critical than mitophagic cargo. This bias is mostly due to the stability and kinetics of the enzymes, rather than lysosomal breakdown or transcriptional reprogramming [130].

Membrane biology influences lysosomal inefficiency. Lipid accumulation alters the composition of lysosomal membranes, impacting their flexibility and curvature. Autophagosome–lysosome fusion requires the formation of SNARE complexes and the presence of certain membrane characteristics [131]. Alterations in lipid content increase the probability of incomplete fusion or delayed cargo delivery, and they also impede fusion kinetics. These delays increase the probability that impaired mitochondria will reintegrate into the functional network by remaining in the cytoplasm for an extended duration. In this instance, redox signalling ceases to be the primary catalyst and transforms into a secondary enhancer. Redox-active intermediates may persist due to the insufficient turnover rate of mitochondria and the inadequate degradation rate of lysosomes. This alters signalling proteins and further diminishes the efficacy of quality-control systems. However, due to their variability and specificity to certain contexts, these alterations introduce noise into regulatory circuits rather than eliciting uniform stress responses [132].

The molecular dysfunctions in oocytes are exacerbated by inherent constraints in organelle dilution and replenishment. When cells fail to divide, damaged components cannot be relocated, and diffusion-mediated restoration is constrained by the extensive cytoplasmic volume. Minor deficiencies in lysosomal and mitochondrial quality control can significantly impair functionality, particularly during energy-demanding activities such as meiotic spindle formation and calcium signalling during fertilisation.

### 8.3. Ovarian Ageing: Progressive Loss of Axis Plasticity Through Molecular Memory and Kinetic Failure

Ovarian ageing signifies a distinct disturbance of the mitochondria–lysosome redox axis, contrasting with hormonally induced or metabolically stressed states. Ageing is defined not by sudden saturation or dominant signalling, but by a gradual, cumulative reduction in adaptive capacity resulting from molecular alterations that increasingly impair the reversibility of quality-control mechanisms. The defining pathogenic characteristic of the disorder is the buildup of molecular memory that restricts the axis to suboptimal operational states, rather than just the existence of damage [133].

With advancing age, the arrangement of the nucleoid and the fidelity of mitochondrial DNA replication gradually alter. These alterations exert a minor yet enduring impact on pulmonary function. Replication mistakes, deletions, and variations in copy counts do not uniformly impact all mitochondrial populations [134]. Rather, they induce variations in transcriptional output and protein stoichiometry. This heterogeneity diminishes the consistency of electron transport efficiency throughout the mitochondrial network by disrupting the exact assembly kinetics of respiratory complexes and supercomplexes. Significantly, these alterations never exceed the thresholds required for acute depolarisation, indicating that dysfunctional mitochondria might persist without activating standard clearance systems [127].

The declining effectiveness of protease-driven degradation and chaperone-assisted folding worsens mitochondrial proteostasis in aged oocytes. Oxidation and carbonylation are two age-related modifications that occur in mitochondrial proteases post-synthesis. These modifications render them less discerning and impede their processing speed. Consequently, broken or improperly folded proteins gradually accumulate within the inner membrane and mitochondrial matrix. These proteins enhance mitochondria’s capacity for energy storage but impair their signalling efficacy by disrupting metabolite flux and enzyme kinetics [135].

The temporal disjunction between damage buildup and quality-control activation is a crucial factor in age-related axis disruption. Temporary stress swiftly triggers mitophagy in young cells, restoring mitochondrial populations before damage accumulates [136]. Damage in old oocytes may endure for a longer period before chemical stabilisation, arising from permanent changes due to delayed recognition and prolonged execution of mitophagy. The subsequent activation of quality control is reduced as these stabilised lesions no longer serve as effective stimuli for adaptive responses [137]. The principal factor contributing to lysosomal dysfunction in ovarian ageing is kinetic deceleration, rather than a reduction in capacity. As individuals age, alterations in acidification dynamics, enzyme turnover, and membrane trafficking diminish the functional capacity of lysosomes, despite their continued presence and structural integrity. The v-ATPase’s capacity to maintain stable proton gradients deteriorates with fluctuations in ATP levels and alterations in the composition of its subunits due to ageing. Even little reductions in acidity significantly hinder the functionality of lysosomal hydrolases, particularly those responsible for the degradation of proteins and organelles [129].

The molecular turnover rate of hydrolase in ageing lysosomes also varies. Age-associated alterations in lysosomal enzymes diminish their stability and catalytic efficiency, necessitating their eventual replacement. This results in a lysosomal population replete with long-lived enzymes that are only partially impaired and function at suboptimal velocities [138]. Consequently, autophagic flux diminishes gradually, resulting in the prolonged persistence and enhanced resilience of injured mitochondria. The membrane dynamics further exacerbate lysosomal function in aged cells. Progressive alterations in the lipid composition of the lysosomal membrane diminish its flexibility and fusion capability. Prolonged delays in the fusion of autophagosomes and lysosomes heighten the probability of insufficient cargo processing or recycling. These deficiencies do not directly induce lysosomal failure; nonetheless, they precipitate a sequence of complications that hinder the cell’s ability to expeditiously eliminate damaged organelles, which is crucial for biological function [139].

Redox signalling is essential for the accumulation of irreversible post-translational modifications that disrupt the ageing-related axis. Redox-sensitive residues in kinases, phosphatases, and scaffold proteins experience irreversible changes following prolonged exposure to low-level oxidative events [140]. These modifications affect baseline signalling states by adjusting response thresholds and reducing adaptive signalling cascades. These alterations, which preserve a molecular record of previous stress exposure, cannot be swiftly reversed like redox signalling [141]. The accumulation of molecular memory reduces the dynamic range of the axis. Signals that once induced rapid changes in lysosomes now elicit delayed alterations or none whatsoever. Even under normal circumstances, aged oocytes are more prone to failure than younger ones. The axis rapidly surpasses these thresholds when new stresses, such as hormonal or metabolic alterations, occur. This results in an abrupt decline in function [135].

Moreover, as we age, the integrity of contact sites deteriorates, impeding communication between organelles. The contact areas between mitochondria and lysosomes rely on cytoskeletal anchoring and precise interactions among proteins and lipids. Age-related alterations in these elements impede the mobility of calcium, lipids, and regulatory signals, hence diminishing the efficacy of quality control by destabilising contact sites and reducing signal effectiveness. This disaggregation complicates the differentiation between the lysosomal reaction and mitochondrial distress [84]. The lack of proliferative dilution and the restricted ability of oocytes to repair their organelles intensify these molecular alterations. For decades, damaged components accumulate, and the compensatory mechanisms of somatic cells are mostly unavailable. Although surrounding somatic cells may exhibit some adaptability, the disruption of ageing-related axes at the germ cell level is fundamentally irreversible.

Functionally, aged oocytes with axis failure demonstrate impaired cytoplasmic maturation instead of overt cell death. Fertilisation and the early stages of embryonic development are hindered by minor discrepancies in ATP distribution, calcium sensitivity, and organelle transmission [142]. Morphological assessment frequently overlooks these anomalies, intensifying the disparity between the real reproductive capacity of older women and the indications of ovarian reserve. From a systems theory perspective, ovarian ageing signifies a transition of the control system from flexibility and adaptability to rigidity, characterised by an abundance of memory and diminished responsiveness. The mitochondria–lysosome-redox axis has transitioned from a dynamic quality control system to a constrained system on the verge of failure. This alteration can elucidate the abrupt decline in fertility that occurs when compensating systems are exhausted.

### 8.4. Poor Ovarian Responders: Intrinsic Axis Insufficiency as a Multi-Layer Molecular Constraint

This section is based on three primary levels of restriction: mitochondrial capacity, proteostatic constraints, and lysosomal throughput. The revelation that the axis malfunctions prior to overt damage, at the level of insufficient molecular infrastructure that determines the maximum attainable throughput of mitochondrial remodelling and lysosomal clearance, represents the initial step toward a mechanistic understanding of suboptimal ovarian response [143]. This infrastructure relies on quantitative measurements that serve as strict limits, including lysosomal enzyme delivery, protease, chaperone turnover, protein import capability, and mtDNA replication potential. The system only saturates when folliculogenesis necessitates fast remodelling, and kinetic delays along with queueing effects put limitations on subsequent operations.

The failure to enhance functional mitochondrial populations through mtDNA replication and nucleoid maintenance frequently represents a major obstacle in poor responders. Both the replication-segregation kinetics of nucleoids and the transcriptional activation of nuclear programmes are essential for mitochondrial growth [144,145,146]. Mitochondrial growth is quantitatively suppressed when replication capacity is constrained, even in the absence of upstream signalling (PGC-1α-like programmes). This constraint may stem from the lessened efficacy of the POLG complex, decreased processivity of TWNK (mtDNA helicase), or compromised stability of the mtSSB-supported replisome. This results in larger mitochondria that are less efficient in performing their functions, indicating a diminished stoichiometric alignment between nuclear-encoded partners and mtDNA-encoded subunits [145].

The organisation of the nucleoid is also significant. Altered TFAM-mediated packing modifies the accessibility of mtDNA templates, influencing transcriptional bursts and the start frequency of replication. When nucleoids are too condensed, the commencement of replication is infrequent. When they are insufficiently compacted, template stability diminishes and mistake rates increase [146]. In both scenarios, the outcome is a diminished dynamic range of variations in mtDNA copy number throughout follicular development. This ceiling is crucial because the “quantity” of mitochondria in oocytes encompasses not only the amount of organelles but also their capacity to produce well-constructed respiratory units with consistent transcription of mtDNA.

Suboptimal responders may encounter limitations in TOM/TIM translocase efficiency, despite nuclear programmes attempting to enhance mitochondrial protein levels. During gonadotropin-mediated remodelling and follicular recruitment, the demand for imports significantly increases [147]. In the absence of the TOM complex, ineffective TIM23/TIM22 function, or impaired translocation reliant on membrane potential, precursor proteins accumulate in the cytosol or become trapped at the outer membrane. This induces a state termed “import stress,” resulting in two consequences: it impedes the transport of catalytic subunits to mitochondria and depletes chaperone resources from other proteostatic tasks.

Import stress induces alterations in cellular translation as a reaction to stress, transcending mere logistical challenges. Activation of ISR nodes by eIF2α phosphorylation can decelerate global translation, thereby safeguarding the proteome while impeding biogenesis. In a low-capacity system, the intended biogenesis programme paradoxically inhibits itself, as this protective reaction remains perpetually engaged during stimulation [148]. This clarifies the mechanical explanation for the “diminishing returns” phenomenon in aggressive stimulation: heightened demand intensifies import stress, resulting in the activation of translational brakes and further limiting mitochondrial remodelling.

Mitochondria require equilibrium among folding (mtHSP70/HSPA9 systems), refolding, and degradation (LONP1, CLPP/CLPX) to maintain functional enzyme complexes [149]. In poor responders, throughput may constrain the axis more significantly than baseline function. When the load is stationary, chaperones and proteases function effectively. But, as metabolic flow and assembly requirements increase, they are unable to rectify faults promptly. Durable misfolded matrix proteins and incompletely formed inner membrane complexes interact non-specifically with catalytic cores, obstructing the access of cofactors [149].

Prolonged error resolution results in maladaptive activation of the mitochondrial unfolded protein response (UPRmt) [5,150]. The oocyte and peri-oocyte environment exhibit constrained and protracted transcriptional responses. UPRmt is designed to restore homeostasis by elevating the amounts of proteostasis components [150]. As a result, the system undergoes a phase of proteostatic lag, wherein errors accumulate at a rate surpassing their rectification, resulting in mitochondria that remain functional yet display diminished catalytic accuracy. This condition variable mitochondria producing unpredictable energy microdomains rather than “nonfunctional mitochondria” compromises oocyte competence [5].

The cytosolic and mitochondrial acetylome regulate enzyme activity in a nuanced yet significant manner in individuals with poor responsiveness [151,152]. The presence of acetyl-CoA and NAD^+^-dependent deacetylases (sirtuins) regulates lysine acetylation, hence altering the functionality of numerous metabolic enzymes and respiratory proteins [151]. Deacetylation facilitated by sirtuins is hindered when NAD^+^ availability is constrained, either due to ineffective mitochondrial NAD^+^ transport, modified salvage pathway flow (NAMPT restriction), or prolonged PARP activation. Enzymes remain present; however, their kinetic parameters alter, resulting in diminished efficiency and reduced coupling, hence affecting the catalytic state of the entire system [152].

This is an issue of “quality,” not “quantity.” It diminishes the margin by which mitochondria can enhance production under stress, hence contributing to the low-reserve phenotype. The NADH/NAD^+^ ratios influence electron entry into cells and the functionality of the antioxidant system, hence impacting redox signals [153]. When the axis is unable to promptly recalibrate enzymatic activity states, it restricts the incidence of poor responders, resulting in scaling failure rather than catastrophic dysfunction. The autophagy machinery may be functional yet inadequately triggered in response to heightened demand, which is a crucial differentiation between poor responders and other infertility phenotypes. Autophagic flow depends on initiation mechanisms, including Beclin1-VPS34 nucleation and ULK1 complex activation, as well as lysosomal degradation [154]. If upstream initiation is deficient, the cell cannot enhance autophagic throughput, regardless of lysosomal functionality. This may be due to AMPK’s limited responsiveness, excessive phosphorylation of ULK1, or insufficient availability of nucleation lipids.

This presents a significant issue during stimulation in systems with limited capacity. The cell elevates the demand for organelle remodelling and metabolic burden, although the initiation of autophagy remains at its baseline level. Damaged or malfunctioning mitochondria persist due to improper cargo transport to the degradative pathway, rather than lysosomal dysfunction [155]. This distinction is significant as it implies that treatments targeting only lysosomes may be ineffective unless the initiation gates are also repaired. When lysosomal capacity is constrained, the delivery and renewal of enzymes frequently constitute the barrier, rather than acidification alone. Proper tagging and translocation of lysosomal hydrolases is essential. This was previously accomplished via mannose-6-phosphate pathways and associated receptors. Lysosomes contain enduring pools of enzymes that initially function effectively. Their growth is impeded if enzyme routing is compromised. In poor responders, the replenishment of tiny hydrolases is ineffective, resulting in diminished adaptive gain, as lysosomes are unable to rapidly increase the concentration of active enzymes in response to more cargo [156].

During periods of significant fluctuation, the turnover of lysosomal membrane proteins, encompassing transporters and other proteins essential for acidification and substrate export, becomes critically crucial [157]. When turnover is sluggish, luminous crowding increases, resulting in restricted product export [157]. This diminishes effective throughput by creating “traffic jams” in degradation, where cargo is disassembled but not efficiently eliminated from lysosomes. Queueing-like effects may manifest in microscopy even in the absence of distinct lysosomal pathology. For oocyte competency, signals must be coordinated. An axis with little capacity typically experiences failures in synchronisation rather than in overall functionality. Contact sites between mitochondria and other organelles facilitate the coordination of calcium and metabolite exchange [158]. Mitochondria are unable to receive calcium pulses at the appropriate intervals to initiate ATP bursts and activate dehydrogenase due to the insufficient responsiveness and stability of the contact sites. ATP and calcium signals must be meticulously synchronised during meiosis. This results in a dissonance between the supply and demand for energy. Synchronisation issues in underperforming responders may manifest as normal baseline energetics that fail under high demand. The system functions “adequately” in silence, although it becomes unstable when subjected to the rapid, throbbing signalling patterns of maturation and activation. This is one reason why phenotyping reliant on static biomarkers may not accurately reflect the severity of malfunction [159].

Ultimately, inadequate buffering at the somatic–germline interface is prevalent among poor responders, as the follicle functions as a singular metabolic entity. The oocyte receives substrates from the granulosa via gap junctions. The substrates comprise pyruvate, amino acids, and redox equivalents. If the granulosa cells operate near their metabolic thresholds, this delivery may alter. Low-reserve restrictions in germ cells are exacerbated by slight variations in substrate delivery, which modify the mitochondrial set points and redox equilibrium of the oocyte. The coupling difficulties exacerbate when ovarian stimulation is regulated. Granulosa cells undergo steroidogenic demands and fast proliferation due to stimulation, which reduces their buffering capacity. The oocyte faces inherent capacity constraints and an external environment that insufficiently stabilises supply, thus hindering axis performance in manners not consistently associated with follicle count or oestradiol response. The redox-mitochondria–lysosome axis is a stable regulatory system, but disorders related to infertility make it unstable by using different molecular entry points, as Table 2 shows.

Different infertility phenotypes interfere with the mitochondria–lysosome-redox axis by creating different molecular bottlenecks. The initiating failure node governs reversibility, adaptive capacity, and therapeutic responsiveness, while the structural components of the axis are preserved.

These disease-specific patterns offer a valuable framework for comprehending axis dysregulation; however, they necessitate careful interpretation. Most of the data that is out there comes from indirect evidence, experimental models, or surrogate cellular systems. There are still not many human studies that directly link these molecular changes to reproductive outcomes, and causal relationships are often assumed instead of shown.

## 9. Discussion

Our review consolidates a significant and diverse body of literature to support the assertion that female infertility is more precisely defined as a disorder of coordinated organelle quality control, rather than simply a result of mitochondrial dysfunction, oxidative stress, or altered autophagy. The ability of ovarian cells to dynamically integrate redox signals, mitochondrial remodelling, and lysosomal degradation into adaptive responses that maintain oocyte integrity throughout time is a consistent feature documented in human studies, animal models, and mechanistic in vitro research. Previous evaluations primarily utilised a parallel approach concerning these processes. Harrath et al. and Nakashima et al. defined autophagy as a conserved homeostatic process influencing folliculogenesis, implantation, and reproductive ageing, while acknowledging significant deficiencies in our comprehension of the particular effects of autophagic flux on oocyte competence [160,161]. Tang et al. underscored the context-dependent duality of autophagy in the ovary, illustrating that pathological diseases such polycystic ovary syndrome, early ovarian insufficiency, and decreased ovarian reserve may arise from both excessive and insufficient autophagic activity [128]. These essential discoveries identify autophagy as a vital yet carefully regulated process, whose influence on reproduction cannot be understood apart from downstream degradative pathways or upstream stress signals. From a clinical perspective, these pathways suggest potential treatment targets. Interventions aimed at reducing oxidative stress or improving mitochondrial quality control have attracted increased attention in reproductive medicine. Researchers have investigated antioxidant substances such as coenzyme Q10, melatonin, and resveratrol to determine their efficacy in enhancing mitochondrial function and reducing oxidative damage in oocytes and granulosa cells.

Oxidative stress is a prevalent upstream factor contributing to infertility, although its effects extend beyond mere cell death. Begum et al. offered a thorough explanation of how spindle abnormalities, meiotic errors, DNA damage, telomere shortening, and follicular atresia exemplify the mechanisms through which reactive oxygen species from mitochondria, granulosa cells, environmental factors, and assisted reproductive technologies affect oocyte quality [77]. This study demonstrated that oxidative stress occurs both in vitro and in vivo. It linked iatrogenic variables associated with embryo culture and ovarian stimulation to inherent ovarian disease. Liu et al. experimentally confirmed the concept that high oxidative stress impairs folliculogenesis and fertility by synchronising the activation of autophagy and apoptosis pathways, using silica nanoparticle exposure models [75]. Their research has shown that oxidative stress not only harms cells but also alters intercellular communication by inhibiting Nrf2/HO-1 signalling and activating the PI3K/AKT/mTOR and PINK1/Parkin pathways. These findings indicate that oxidative stress should not be viewed as a definitive injury but rather as a signalling input for appropriate downstream resolution.

Mitochondrial-focused research clarifies this viewpoint, showing that infertility frequently occurs with mitochondrial malfunction despite no substantial decrease in mitochondrial mass. Ma et al. and Ju et al. suggest that mitochondrial failure is a defining marker of ovarian ageing [23,162]. They highlighted that a decreased mtDNA copy number, altered mitochondrial dynamics, and compromised bioenergetics synergistically result in a decline in oocyte quantity and quality. When evaluated in conjunction with the findings of Cox et al., Cota et al., and Shen et al., who collectively established that the failure to maintain mitochondrial quality, rather than quantity, is the principal determinant of reproductive ageing, these results gain greater mechanistic significance [50,137,163]. Cota et al. presented interspecies evidence demonstrating that the maintenance of mitochondrial turnover through mitophagy is crucial for prolonged reproductive longevity, while Cox et al. showed that the age-related decline in oocyte competence correlates with the accumulation of mtDNA mutations and impaired mitochondrial quality control [137,163]. Shen et al. established that, although mitophagy regulators are present in mature oocytes, mitophagy is primarily inhibited in these cells [50]. Mitophagy actively contributes to early oogenesis and granulosa cells, promoting mitochondrial remodelling and oocyte development. This selective suppression may impede developmental competence during a crucial phase of vulnerability when faulty mitochondria can persist and propagate to embryos.

The lysosomal system is a critical constraint that influences the body’s capacity to rectify mitochondrial and redox issues. Yamada et al. established that the age-associated buildup of the autophagy inhibitor Rubicon obstructs autophagic flux in granulosa cells, resulting in the accumulation of metabolic enzymes including ATP citrate lyase, a reduction in oxidative stress resilience, and a decline in oocyte quality [164]. These findings clarify a molecular connection between metabolic dysregulation, reproductive ageing, and compromised autophagy. Tang et al. further substantiated the notion that the decline in degradative ability is a pivotal factor in ovarian ageing [128]. Lysosomal insufficiency results in diminished ovarian reserve and premature ovarian insufficiency. Zhou et al. demonstrated a relationship between oxidative stress and lysosomal dysfunction by revealing that advanced oxidation protein products disrupt autophagy–lysosome pathways via ROS-dependent mTOR activation and inhibition of TFEB nuclear translocation, thereby impeding lysosomal biogenesis [56]. These processes illustrated that chronic oxidative stress might provoke persistent lysosomal insufficiency, impeding cellular recovery, and were corroborated in both in vitro granulosa cell models and in vivo rat ovaries.

Mitophagy is crucial at the intersection of the lysosomal and mitochondrial pathways. It appears to be a significant factor in the competence of oocytes in both healthy and diseased individuals. Mitophagy is rigorously regulated during follicular development, promoting mitochondrial quality control in the early stages while being reduced in mature oocytes, as evidenced by Zhou et al. and Shen et al. Cox et al. established a connection between mitochondrial clearance failure and compromised embryonic development by arguing that inadequate mitophagy during oocyte ageing promotes the accumulation and transgenerational transmission of defective mitochondria [50,56,137]. Cota et al. presented persuasive functional evidence demonstrating that the enhancement of mitophagy through genetic or pharmacological means protects oocyte quality and prolongs reproductive longevity [163]. Genetic studies indicating that the disruption of mitophagy regulators results in significant reproductive consequences further validate these findings. Deficiency in mitophagy was directly linked to reproductive failure in Parkin knockout animals, which demonstrated decreased fertility across multiple age cohorts, reduced oocyte production, and impaired fertilisation rates.

Research focused on specific diseases indicates that infertility phenotypes predominantly vary in the causes and timing of axis disruption, rather than in the particular pathways involved. Kobayashi et al. revealed that increased testosterone levels in polycystic ovarian syndrome modify mitochondrial dynamics, metabolism, and mitophagy in granulosa cells and oocytes [47]. Their research indicated that a transient transition to mitochondrial fission maintains quality control. However, prolonged fission halts autophagy and mitophagy due to the depletion of the compensating reserve, initiating apoptosis. Harrath et al. and Tang et al. contend that the aetiology of PCOS is influenced by both excessive and inadequate autophagy, indicating instability in quality-control regulation rather than uniform suppression [128,160]. These data emphasise that PCOS constitutes a state of compensatory vulnerability and signalling bias, rather than permanent organelle damage.

Oxidative stress appears to be a significant contributor to axis saturation in infertility models associated with obesity and pollutants. Liu et al. experimentally established that extended exposure to oxidative stress leads to concurrent activation of autophagy and death, rather than adaptive remodelling [75]. Simultaneously, Begum et al. recognised obesity as a substantial factor influencing oxidative stress that affects oocyte quality [77]. The data suggest that cells adopt maladaptive quality-control states when extended metabolic excess surpasses mitochondrial and lysosomal capability. In contrast, ovarian ageing is marked by a progressive reduction in adaptive plasticity rather than sudden saturation. Research by Ma et al., Ju et al., Yamada et al., Cox et al., and Tang et al. collectively indicates that, even without explicit stress, ageing is marked by a progressive accumulation of irreversible molecular changes, diminished lysosomal function, and an impaired ability to activate effective autophagic responses [6,128,137,162,164].

Interventional studies rigorously support this integrated axis model, highlighting its translational importance. Gui et al. shown that pharmacological activation of mitophagy through HEP14 reinstates ovarian endocrine function and follicular regeneration in elderly mice by increasing PKC-ERK1/2 signalling, improving mitochondrial quality control, and diminishing oxidative stress [165]. Restoring mitochondrial-lysosomal coordination can reverse functional decline, since transcriptome investigations demonstrate molecular rejuvenation patterns similar to those found in younger ovaries. Cota et al. demonstrated that urolithin A-induced mitophagy enhances reproductive longevity by preserving youthful mitochondrial structure and turnover [163]. The causal link between mitochondrial clearance capacity and reproductive health was further validated by the experimental inhibition of mitophagy through genetic deletion of Parkin or chronic suppression of lysosomal biogenesis, which consistently led to reduced fertility and impaired oocyte competence.

Infertility results from the disruption of oxidative stress signalling, mitochondrial remodelling, and lysosomal degradation, according to a unified paradigm supported by the studies reviewed in this context. Autophagy and mitophagy function as interconnected defensive mechanisms [166]. Rather, they are interrelated processes whose efficacy is influenced by lysosomal capacity, metabolic demand, and redox state. Any disruption within this network, whether due to signalling bias, degradative saturation, or intrinsic capacity constraints, might result in poor decisions regarding organelle fate and a gradual deterioration in oocyte quality. In vulnerable populations, intense ovarian stimulation paradoxically worsens outcomes by increasing metabolic demand without restoring quality-control mechanisms.

Static indicators of oxidative stress, mitochondrial mass, or autophagy related protein expression may fail to capture the dynamic functionality of the mitochondria–lysosome-redox axis. Future study should focus on the functional evaluations of ovarian cells’ ability to respond to stress, reorganise organelles, and restore homeostasis. These technologies may facilitate more accurate patient categorisation and the creation of focused therapies that restore coordination instead of merely suppressing individual abnormal signals. From a translational point of view, it might be better to restore axis synchronisation instead of focusing on isolated molecular nodes (Table 3).

Therapeutic approaches for female infertility are progressively concentrating on the redox-mitochondria–lysosome axis components. For interventions to be effective, it is probable that lysosomal degradation capacity, redox thresholds, and mitochondrial signalling must be reinstated in a synchronised manner.

The identification of the redox-mitochondria–lysosome axis also generates novel therapeutic opportunities. Alongside conventional antioxidant supplements, there is increasing interest in approaches that emphasise mitochondrial metabolism and organelle quality management. Metabolic therapies aimed at improving mitochondrial bioenergetics, including the control of NAD^+^ metabolism and the activation of AMPK, have been proposed as effective ways to restore cellular homeostasis in the ovarian environment. Furthermore, experimental studies suggest that pharmacological agents targeting autophagy and mitophagy may provide a potential therapeutic pathway. These techniques might potentially enhance oocyte competence and reproductive outcomes by facilitating the elimination of defective mitochondria and reinstating lysosomal function. Although the majority of these techniques remain in the experimental phase, they underscore the potential significance of targeting organelle quality control mechanisms in assisted reproduction.

A key shortcoming of the present synthesis is that the suggested axis is not generated from a solitary experimental system but rather from the amalgamation of data across several biological models. Consequently, it must be regarded as a conceptual framework requiring specific experimental confirmation in human reproductive tissues. The substantial heterogeneity observed among patients with analogous clinical diagnoses may also be influenced by these mechanistic patterns. Even within the same diagnostic category, variations in ovarian response, oocyte quality, and treatment outcomes may be attributable to variations in the extent and severity of axis disruption.

## 10. Limitations and Future Research

Notwithstanding the integrative approach presented in this study, it is crucial to recognise certain notable limitations that also define research goals. A substantial amount of evidence linking female infertility to mitochondrial malfunction, autophagy, lysosomal competence, and redox imbalance originates from in vitro systems or animal models. These may not precisely reflect the cellular variety and temporal intricacy of the human ovary, although they offer mechanistic insights. Many investigations rely on static markers that inadequately reflect dynamic quality-control capacity, as directly assessing autophagic and mitophagic flux in human oocytes and granulosa cells presents significant technical challenges. The comparative importance of pathways mediated by somatic cells versus oocytes is unclear, particularly regarding ageing and inadequate ovarian response, as systemic metabolic and inflammatory variables may exacerbate organelle coordination disruption. Thus, prioritising the development of functional assays to evaluate lysosomal degradative capability, mitochondrial turnover, and redox adaptation in human reproductive tissues, ideally within a longitudinal framework, should be the primary focus of future research. The combination of live-cell imaging, metabolic flow analysis, and sophisticated single-cell and spatial omics techniques may clarify changes in organelle communication networks over the reproductive lifespan and in various clinical conditions. To enhance the precision of therapeutic interventions in female infertility, interventional studies in assisted reproduction should assess targeted methodologies focused on restoring organelle coordination and adaptive capacity, rather than utilising generic antioxidant or stimulation-based strategies.

The discipline is significantly limited by its reliance on experimental models that do not adequately capture the complexities of human follicular biology. It is even more challenging to comprehend the situation due to the variety of stimulation protocols, patient groups, and methods applied. It will be essential to conduct subsequent investigations that incorporate high-resolution molecular data from human reproductive tissues in order to verify and improve the proposed mechanisms.

## 11. Conclusions

This narrative review indicates that female infertility results from a disruption in coordinated organelle quality control, rather than from isolated deficiencies in autophagic activity, oxidative stress balance, or mitochondrial function. This study emphasises the mitochondria–lysosome-redox axis as a dynamic regulatory system that controls cellular adaptation, oocyte competence, and organelle destiny decisions over the reproductive lifespan, synthesising insights from experimental, translational, and clinical research. The literature assessment suggests that autophagy and mitophagy function as context-dependent integrators rather than generally protective systems. Moreover, oxidative stress is both as a source of cellular damage and a signalling mechanism, with its resolution dependent on proper mitochondrial remodelling and lysosomal degradation capacity. Different forms of infertility, such as polycystic ovary syndrome, obesity-related infertility, ovarian ageing, and poor ovarian response, represent distinct failures of the axis, encompassing a gradual decline in adaptive plasticity, intrinsic capacity limitations, signalling bias, and saturation of degradation. Understanding these variances highlights the need for more targeted, mechanism-based therapies and offers a mechanistic explanation for the limited effectiveness of traditional therapeutic approaches like antioxidant supplementation or increased stimulation. Future progress in reproductive medicine will likely hinge on the ability to evaluate and restore organelle coordination and adaptive capacity, rather than solely addressing the downstream symptoms of failure. Examining female infertility via the perspective of integrated organelle quality management provides a conceptual basis for improved patient classification, focused interventions, and eventually more effective and personalised assisted reproductive therapies.

Translationally, the proposed model provides a prospective framework for transitioning from a purely descriptive clinical marker system to a more mechanism-based stratification of patients. In order to more accurately predict reproductive outcomes and assist physicians in developing personalised treatment plans, future methodologies may incorporate indicators of mitochondrial function, redox balance, and lysosomal competence in addition to markers of ovarian reserve or hormonal profiles.

## Figures and Tables

**Table 1:** Core molecular components of the redox-mitochondria–lysosome axis in female reproductive cells.

Molecular Mediator	Biological Function	Role in Oocytes	Role in Granulosa Cells	Type of Evidence
NRF2/KEAP1	Regulation of antioxidant response and redox homeostasis	Maintains intracellular redox balance during meiotic arrest	Activates antioxidant defence and supports steroidogenesis	Animal/in vitro
Sirtuins (SIRT1, SIRT3)	NAD^+^-dependent regulation of mitochondrial metabolism and oxidative stress	Supports mitochondrial function and meiotic competence	Regulates metabolic adaptation and ROS detoxification	Animal/in vitro
DRP1	Mitochondrial fission and segregation of damaged organelles	Enables removal of dysfunctional mitochondrial fragments	Regulates mitochondrial turnover under metabolic stress	Animal/in vitro
MFN1/2, OPA1	Mitochondrial fusion and network integrity	Maintains mitochondrial distribution and energy balance	Supports mitochondrial connectivity and steroidogenic function	Animal/in vitro
PINK1/Parkin	Ubiquitin-mediated mitophagy initiation	Marks depolarized mitochondria for degradation	Facilitates mitochondrial quality control under stress	Animal/in vitro
BNIP3/NIX/FUNDC1	Receptor-mediated mitophagy (hypoxia/metabolic stress response)	Regulates mitochondrial turnover during metabolic shifts	Supports granulosa cell differentiation and adaptation	Animal/in vitro
ULK1/Beclin1-VPS34	Autophagosome formation and initiation of autophagy	Enables sequestration of damaged mitochondria	Maintains autophagic flux under stress conditions	Animal/in vitro
TFEB/CLEAR network	Regulation of lysosomal biogenesis and autophagy genes	Controls lysosomal capacity for long-term organelle turnover	Supports adaptive lysosomal response to metabolic demand	Animal/in vitro
v-ATPase	Lysosomal acidification and enzymatic activation	Ensures degradation of autophagic cargo	Maintains lysosomal function under sustained metabolic load	Animal/in vitro
Cathepsins	Lysosomal proteolysis and degradation of organelles	Degrades mitochondrial remnants	Supports intracellular recycling and metabolic adaptation	Animal/in vitro
Mitochondria–Lysosome Contact Proteins	Organelle communication (Ca^2+^, lipid exchange)	Coordinates mitochondrial signalling and calcium dynamics	Regulates metabolic signalling and stress adaptation	In vitro/indirect

**Table 2:** Disease-specific molecular patterns of axis disruption in female infertility.

Clinical Condition	Primary Molecular Entry Point	Mitochondrial Alteration	Lysosomal Alteration	System-Level Effect
PCOS	Androgen-driven mTOR bias	Fragmented but viable mitochondria	Signalling-driven TFEB suppression	Signal misinterpretation
Obesity	Nutrient saturation	Supercomplex instability	Lysosomal workload saturation	Decision fidelity loss
Ovarian Ageing	Molecular memory accumulation	mtDNA heterogeneity	Kinetic slowing of degradation	Axis rigidity
Poor Responders	Limited biogenetic throughput	Import stress, proteostatic lag	Insufficient adaptive scaling	Capacity ceiling

**Table 3:** Therapeutic strategies targeting the redox–mitochondria–lysosome axis.

Therapeutic Strategy	Primary Target	Mechanistic Rationale	Limitations
Antioxidants	ROS buffering	Reduce redox noise	Do not restore lysosomal capacity
mTOR Modulators	TFEB activation	Enhance lysosomal biogenesis	Context-dependent effects
Mitophagy Activators	PINK1/Parkin pathway	Improve mitochondrial clearance	Requires intact lysosomes
NAD^+^ Boosters	Sirtuin activation	Restore redox-metabolic coupling	Limited in advanced ageing
Metabolic Optimisation	Substrate modulation	Reduce mitochondrial overload	Slow response kinetics

## Data Availability

No new data were created or analyzed in this study. Data sharing is not applicable to this article.
